# An Unusual Presentation of Acute Abdomen Pain: Splenic and Renal Emboli from Left Ventricular Thrombus

**DOI:** 10.7759/cureus.2509

**Published:** 2018-04-19

**Authors:** Kyawzaw Lin, Aung Naing Lin, Won Jun Park, Pwint Phyu Hlaing, Cesar Ayala-Rodriguez

**Affiliations:** 1 Internal Medicine, Brooklyn Hospital Center/Mount Sinai Hospital, New York, USA; 2 Cardiology Fellow, Cardiology Department, Academic Affiliate of the Icahn School of Medicine at Mount Sinai Clinical Affiliate of the Mount Sinai Hospital, New York, USA; 3 University of Medicine(i), Yangon,myanmar; 4 Cardiology Attending, Cardiology Department, Academic Affiliate of the Icahn School of Medicine at Mount Sinai Clinical Affiliate of the Mount Sinai Hospital, New York, USA

**Keywords:** splenic infarction, renal infarction, left ventricular thrombus, left ventricular aneurysm, ischemic cardiomyopathy, acute abdomen pain, gastroenterology, cardiology, general surgery

## Abstract

Splenic infarction is an unusual cause for a patient to present with left upper abdomen pain. A 47-year-old woman presented to the emergency department with left upper abdomen pain. She reported that she stopped taking warfarin two days prior to presentation. A physical examination revealed fine crackles within the left lower lobe and significant tenderness within the left upper abdomen. Computed tomography of the abdomen showed mild cardiomegaly with a 2.3 cm calcified thrombus in the left ventricular apex. We noted infarction in the spleen and right kidney with bilateral renal scarring. The patient was initially started on a heparin drip and later bridged to warfarin on the third day. She was discharged after seven days with complete resolution of the abdominal pain. The decision to prescribe an anticoagulant should include a consideration of underlying causes, comorbidities, an assessment of risks and benefits, and chances of recurrence. In our patient, her new splenic infarct and renal infarction were most likely embolic in origin due to her left ventricular apical aneurysm with thrombus and nonadherence to her prescribed anticoagulation medication.

## Introduction

Splenic infarction is usually asymptomatic and found during radiographic examinations for other conditions. We present a case of a patient with concerns of left upper abdominal pain, which were likely due to splenic infarct.

## Case presentation

**Figure 1:**
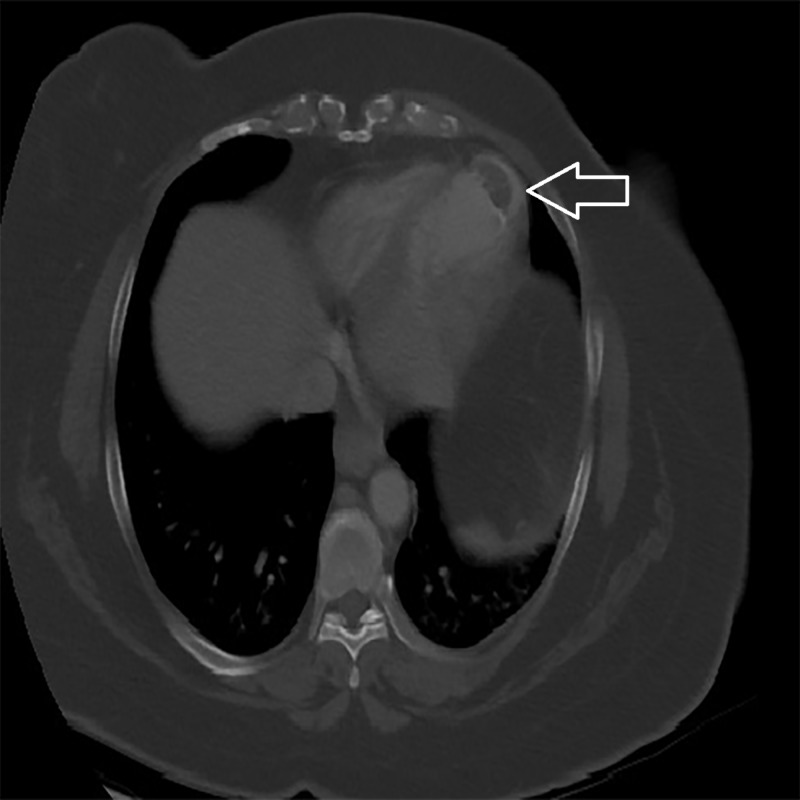
Computed tomography of the abdomen showing mild cardiomegaly, old infarction on the anterior-inferior wall of the left ventricle. There is a 37.47 mm hypodense calcified thrombus along the left ventricular apex (white arrow).

A 47-year-old woman presented to the emergency department with concerns of left upper abdominal pain for one day. She reported that the moderate to severe abdominal pain had a gradual onset, was crampy, constant, and located in the left upper part of her abdomen radiating to the lower chest and back. The pain was associated with diaphoresis, nausea, and several instances of vomiting non-bloody, non-bilious liquid. On admission, her vitals were stable. The results of her complete blood counts, comprehensive metabolic panels, and hepatic function tests were within reference ranges. Her coagulation profile was at a subtherapeutic level. Her past medical history includes nonischemic cardiomyopathy with left ventricular thrombus and four previous cerebrovascular accidents with residual right leg numbness. Her condition was being managed with warfarin. A physical examination showed fine crackles within the left lower lobe and significant tenderness in the left upper abdomen. The patient reported that she had stopped taking warfarin two days prior to admission. Cardiac magnetic resonance imaging (MRI) showed septal and apical predominantly transmural infarct. A transthoracic echocardiogram showed a mildly dilated left ventricle with moderate to severe left ventricular dysfunction, an ejection fraction of 30% to 35% with distal anterior, anteroseptal, and apical akinesis with mild mitral regurgitation. Cardiac catheterization findings were unremarkable. A computed tomography (CT) scan of her abdomen showed mild cardiomegaly with previous infarction on the anterior-inferior wall of the left ventricle. We noted a 37.47 mm calcified thrombus in the left ventricular apex with peripheral calcifications (Figure 1).

**Figure 2:**
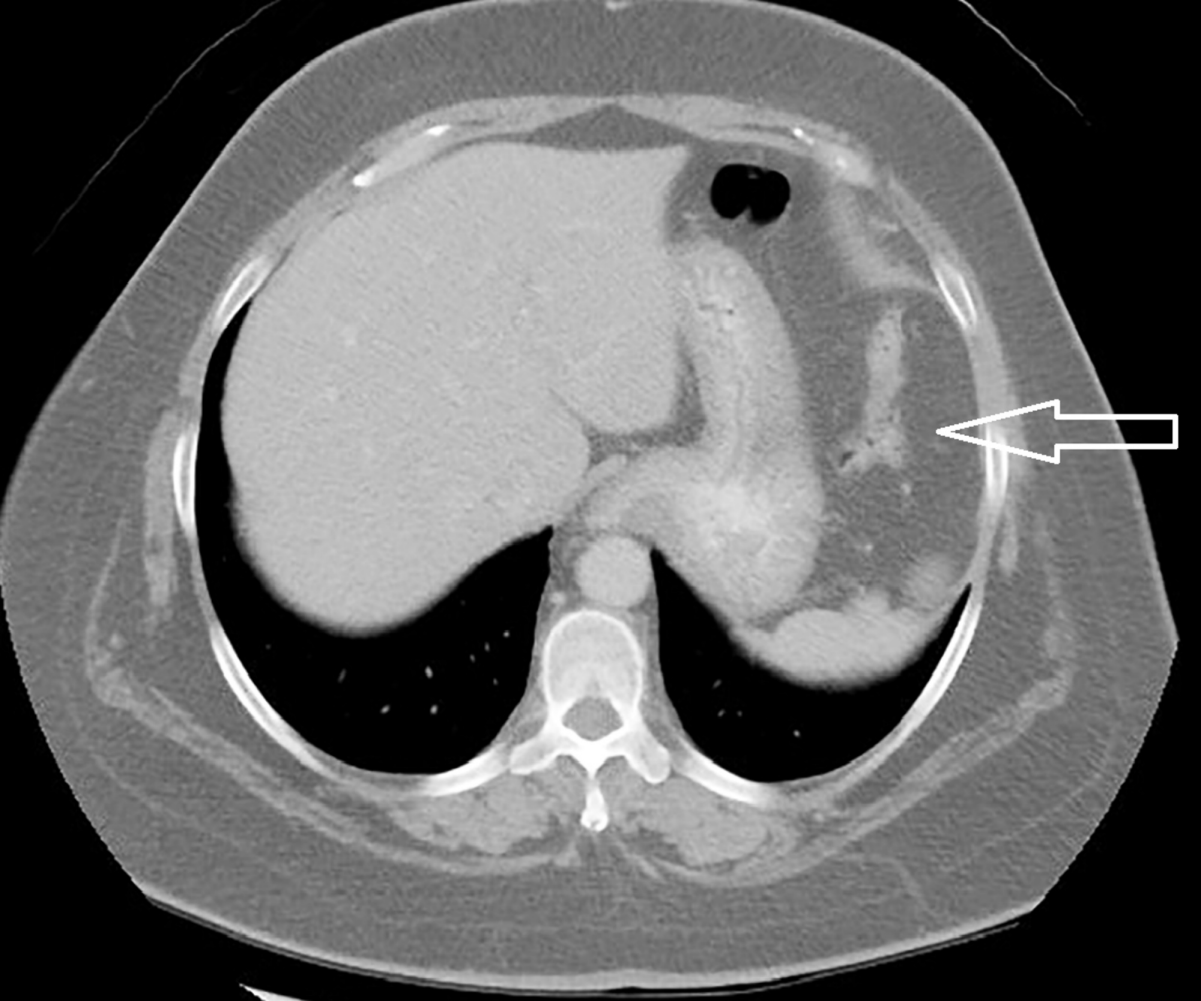
Computed tomography of the abdomen showing a wedge-shaped infarct area measuring 39 mm x 28 mm x 50 mm in the anterior aspect of the spleen (white arrow).

**Figure 3:**
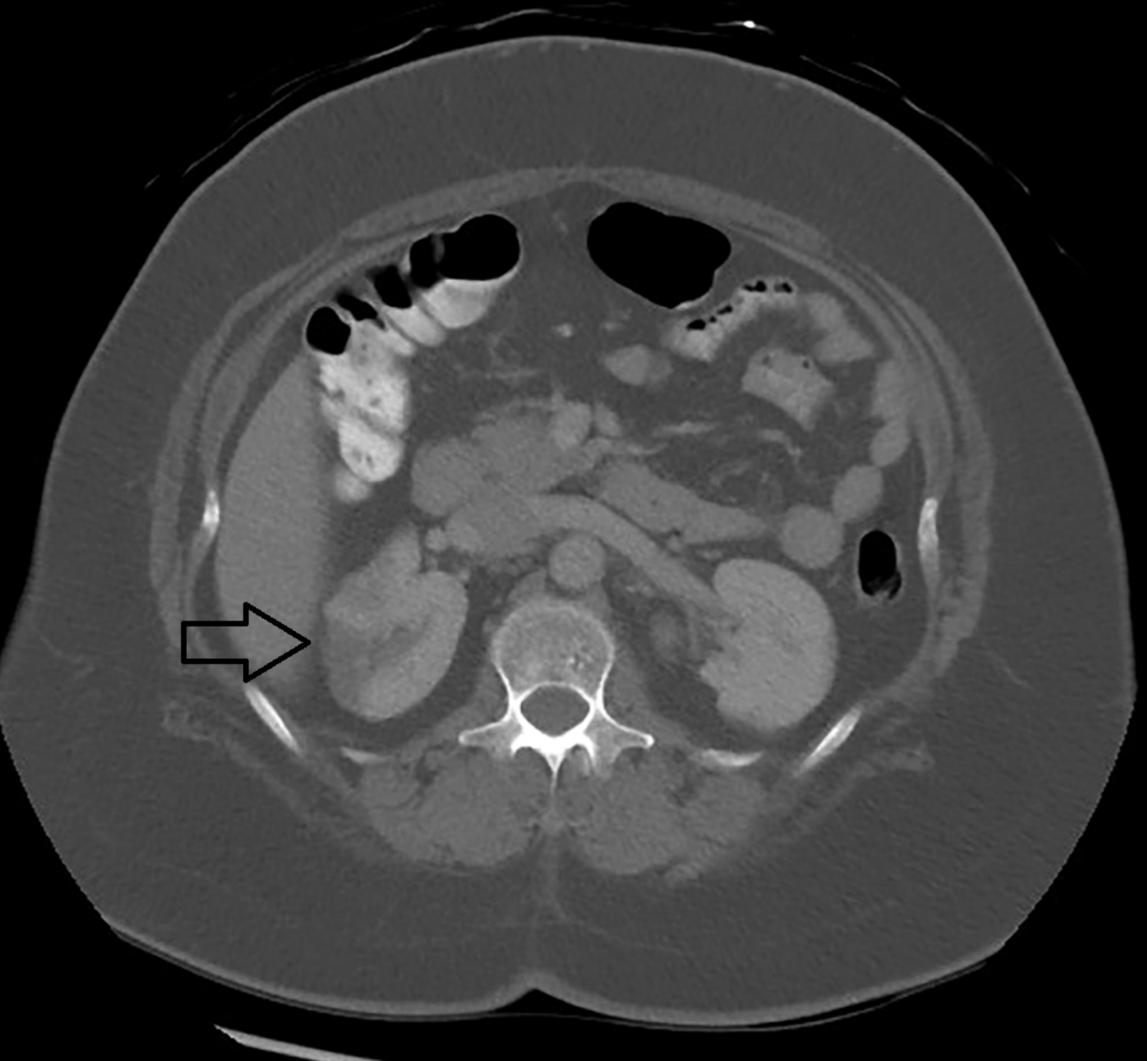
Computed tomography of the abdomen showing an infarct area measuring 42 mm x 22 mm x 27 mm in the upper pole ( dark arrow) and additional subtle hypodensity in the anterior aspect of the midpole of the right kidney. Bilateral renal scarring is present.

We also noted infarction in the spleen and right kidney with bilateral renal scarring (Figures 2-3).

The patient was initially started on a heparin drip and later bridged to warfarin on the third day. She was discharged with complete resolution of her abdominal pain after seven days, and medication compliance was reinforced before discharge.

## Discussion

Splenic infarction is a rare but not uncommon cause of acute abdominal pain in the emergency room. Splenic infarct may occur as a result of occlusion of the arterial or venous supply. The condition can also be caused by infiltrative hematological malignancies such as leukemia, lymphoma, and myelofibrosis. Benign congenital or acquired blood disorders such as sickle cell disorders, polycythemia, and hypercoagulopathies can also result in splenic infarct, as can vasoembolic conditions such as atrial fibrillation, infective endocarditis, valvular heart disease, valve replacement surgery, and aortic atherosclerosis [1].

The most common presenting features of splenic infarct include left upper abdomen pain, fever, left shoulder pain, pleuritic chest pain, back pain, and abdomen distension. Some cases may present with acute abdomen pain from splenic rupture and torsion of the congested spleen. However, most patients with thromboembolic infarction are symptomatic; fever is associated with 70% of cases, and abdomen pain is noted in 80% of cases [1]. However, thromboembolic phenomena only occur in 2% of patients, particularly during the recovery period from acute myocardial infarction [2]. Septic emboli may be the underlying etiology of splenic infarction and splenic abscess in immunocompromised patients. 

Risk factors for infective endocarditis, heart valve replacement, hematological disorders or associated peripheral vascular disease can be obtained from pertinent history and via physical examination. Significant thrombocytosis, high lactate dehydrogenase levels, and elevated erythrocyte sedimentation rates can be found in laboratory tests. CT, nuclear imaging, and sonography are commonly used for diagnostic purposes, but CT scans are the preferred method often used in emergency departments in most institutions. CT imagery can help identify splenic infarction or associated splenic pathologies, although a liver-spleen scan has higher sensitivity in diagnosis. Sonography is used in circumstances where a CT scan cannot be performed and to follow up on complications of splenic infarction. Jaroch reported that a liver-spleen scan could identify 90% of splenic infarctions while only 75% is evident in CT scans. Mesenteric vasculature can be evaluated via MRI [3], and an angiogram can be used if vascular pathology is suspected.

The underlying causes of splenic infarction should be identified to prevent further episodes and complications. A transthoracic echocardiogram should be performed to check for patent foramen ovale as a possible source of emboli, although atherosclerotic disease of the aorta and mesenteric vessels is the most common cause. Consequences of splenic infarction include abscess formation, pseudocyst, infection, and hemorrhage.

The incidence rate of complications may be higher in splenectomies performed for splenomegaly [4]. Conservative management is common for splenic infarction unless it is associated with expanding subcapsular splenic hematoma, pseudocysts, splenic rupture or splenic abscesses. Most patients recover with watchful management and sonographic follow-up to monitor for impending complications. In elderly populations with thromboembolic phenomena, anticoagulation with warfarin or newer oral anticoagulant agents can be considered to prevent further episodes of embolic showering. Vena cava filter placement is another alternative for use in patients with high chances of bleeding or fall, or for patients with recent intracranial bleeding or major surgery.

## Conclusions

Splenic infarction must be considered as a potential cause of acute abdominal pain whenever a patient presents with left upper abdominal pain with or without shock. Splenic infarction can be a complication of the cardiac embolic disease. Most patients respond to conservative management, and the decision to prescribe an anticoagulant should include a consideration of underlying causes, comorbidities, an assessment of risks and benefits, and chances of recurrence. In our patient, the new splenic infarct and renal infarction were most likely due to left ventricular apical aneurysm with thrombus and nonadherence to anticoagulation medication.
